# Methanobactin-Mediated Synthesis of Gold Nanoparticles Supported over Al_2_O_3_ toward an Efficient Catalyst for Glucose Oxidation

**DOI:** 10.3390/ijms151221603

**Published:** 2014-11-25

**Authors:** Jia-Ying Xin, Kai Lin, Yan Wang, Chun-Gu Xia

**Affiliations:** 1 Key Laboratory for Food Science & Engineering, Harbin University of Commerce, Harbin 150076, China; E-Mails: glklkk@126.com (K.L.); wyan@hrbcu.edu.cn (Y.W.); 2 State Key Laboratory for Oxo Synthesis & Selective Oxidation, Lanzhou Institute of Chemical Physics, Chinese Academy of Sciences, Lanzhou 730000, China; E-Mail: cgxia@licp.ac.cn

**Keywords:** methanobactin, bioreduction, glucose oxidation, gold nanoparticles, Au/Al_2_O_3_ catalysts

## Abstract

Methanobactin (Mb) is a copper-binding peptide that appears to function as an agent for copper sequestration and uptake in methanotrophs. Mb can also bind and reduce Au(III) to Au(0). In this paper, Au/Al_2_O_3_ catalysts prepared by a novel incipient wetness-Mb-mediated bioreduction method were used for glucose oxidation. The catalysts were characterized, and the analysis revealed that very small gold nanoparticles with a particle size <4 nm were prepared by the incipient wetness-Mb-mediated bioreduction method, even at 1.0% Au loading (*w*/*w*). The influence of Au loading, calcination temperature and calcination time on the specific activity of Au/Al_2_O_3_ catalysts was systematically investigated. Experimental results showed that decomposing the Mb molecules properly by calcinations can enhance the specific activity of Au/Al_2_O_3_ catalysts, though they acted as reductant and protective agents during the catalyst preparation. Au/Al_2_O_3_ catalysts synthesized by the method exhibited optimum specific activity under operational synthesis conditions of Au loading of 1.0 wt % and calcined at 450 °C for 2 h. The catalysts were reused eight times, without a significant decrease in specific activity. To our knowledge, this is the first attempt at the preparation of Au/Al_2_O_3_ catalysts by Mb-mediated *in situ* synthesis of gold nanoparticles.

## 1. Introduction

Gluconic acid is an important chemical intermediate, which is used extensively as a chelating agent for cleaning applications or as an additive in food and beverages [1,2]. Currently, the production of gluconic acid is based on microbial fermentation and biochemical transformation, which is complicated by the large amount of waste water produced [2]. Synthesis of gluconic acid with catalytic oxidation methods in heterogeneous catalytic systems using supported noble metal nanoparticles as catalysts is a good choice. In these noble metal catalysts, gold catalysts exhibited extremely high catalytic activity for glucose oxidation, which was far superior to the conventional catalysts containing platinum or palladium in terms of activity and 100% selectivity towards gluconic acid [3]. This makes supported gold catalysts the most likely candidates for the replacement of biocatalysts in industrial glucose oxidation. Nowadays, supported gold nanoparticles (GNPs) have been used as highly active catalyst for the oxidation of glucose [4,5,6,7,8].

It has been widely accepted that the catalytic performance of supported gold catalysts is dependent on the particle size of GNPs, the dispersion of GNPs, the interaction between GNPs and supports and the characteristics of the supports [9,10]. The size of the particle appears to be the most important parameter with respect to activity and selectivity in reactions catalyzed by gold. As in the case of GNPs deposited on metal oxides, obtaining small GNPs with a narrow size distribution is considered to be one of the most important targets. Today, several preparation methods are used to generate catalysts with small and active gold particles [3,11,12,13]. The most frequently used preparation methods for depositing Au as nanoparticles on a support include co-precipitation, deposition-precipitation, impregnation, *etc.*

Co-precipitation is the simplest method by which a solid is obtained for the supported metallic GNPs, and it is necessary to use the calcination and/or reduction procedures. Unfortunately, the sintering and redistribution of the Au species occurs during the calcination and reduction procedures, which results in the formation of large metallic Au particles with a regular morphology and of low energy (electronically very stable) [14].

The deposition-precipitation method has the advantage of passing on hydrogen for reduction. However, the deposition-precipitation route has numerous variable parameters, which results in poor reproducibility. In addition, the Au capture efficiency is always very low (<60%), and the amount of waste water is always high [15].

With regard to a technical preparation process, the conventional impregnation method seems to be most feasible [16,17]. However, catalysts produced from the conventional impregnation method usually have a poor GNPs dispersion level. One reason is that Au has a lower melting point and a lower affinity for metal oxides than Pd and Pt. Another reason is that during calcination of HAuCl_4_ crystallites, which are dispersed on the support surfaces, chloride ions markedly enhance the coagulation of gold particles.

From ecologic and economic standpoints, a modified conventional impregnation method, named the incipient wetness method, has some advantages over the deposition-precipitation method, because of no gold loss and no wastewater production. However, the gas-phase reduction using hydrogen is necessary, because chloride present on a gold catalyst promotes the mobility and agglomeration of the gold particles during the calcination procedure, which results in the formation of large gold particles [14].

Although supported nanogold catalysts are routinely synthesized by the above chemical approaches, the biosynthesis method has also become necessary, since the process is more economical and eco-friendly [18]. Much effort has been given toward the biosynthesis of metal nanoparticles in the past decade, and significant advances have been achieved [19]. The biosynthesis method can be used to synthesize stable GNPs with a narrow size distribution and a desired diameter without any auxiliary surfactant or capping agent. Biomolecules can play dual roles as both reductant and stabilizers during the synthesis process. Either microorganisms, such as bacteria [20] and fungus [21], or plant extracts [22] have been employed as a simple, low-cost and eco-friendly approach to synthesize gold nanoparticles. In these methods, synthesis of nanoparticles were triggered by several bioactive compounds such as terpenoids, phenolics, flavonones, proteins, pigments, alkaloids and other reducing agents are present in the microbial cells and plant extracts. However, the exact mechanism of nanoparticle biosynthesis on a cellular and molecular level is yet to be understood. There are few reports to date focusing on the preparation of nano-catalysts employing homogeneous bioactive compounds. A biological process with the ability to strictly control the size and shape of the particles, including the isolation and identification of the compounds responsible for the reduction of gold ions, is still an issue and an ongoing area of research.

Methanobactin (Mb) is a copper-binding small peptide that appears to function as an agent for copper sequestration and uptake in methanotrophs. The crystal structure of copper-loaded Mb (Cu-Mb) from *Methylosinus trichosporium* OB3b revealed a 1217-Da molecule with a chemical composition of C_45_N_12_O_14_H_62_S_5_Cu [23]. Mb can coordinate a single Cu (II) ion by its nitrogens from two oxazolone rings and sulfurs from two enethiol groups and then reduce Cu(II) to Cu(I) [24]. Mb can also bind to a number of other metals, including gold, iron, nickel, zinc, cobalt, cadmium, mercury and uranium [25]. It has been found that Au(III) can be reduced to Au(0), and then Au(0) remains associated with the Mb [25]. In our previous work, a facile Mb-mediated one-step synthetic route to prepare monodispersed GNPs has been developed [26].

In the present work, we demonstrate for the first time that it is possible to prepare Au/Al_2_O_3_ catalyst using Mb by the incipient wetness-Mb-mediated bioreduction method. The catalyst preparation parameters were systematically investigated. We have also studied their catalytic performance for the glucose oxidation in aqueous media. The Au/Al_2_O_3_ catalyst showed an excellent specific activity and durability for the glucose oxidation with aqueous H_2_O_2_ (30 wt %) as the oxidant. To the best of our knowledge, this is the first report of the application of Mb-mediated biosynthesized Au/Al_2_O_3_ catalyst for glucose oxidation.

## 2. Results and Discussion

### 2.1. Preparation and Characterizations of Mb-Mediated Bioreduction Au/Al_2_O_3_ Catalyst

The catalytic performance was more significantly affected by the size of GNPs. Gold catalysts prepared by the incipient wetness method are unsuitable, because the resulting gold particles are quite large, even at a low gold content. These chloride-containing gold complexes seem to be responsible for the failure of the incipient wetness method. Several authors [14,16,17] have shown that chloride enhances the mobility and agglomeration of gold species during the calcination process. Therefore, most gold catalysts prepared by the conventional incipient wetness method need further reduction treatment by calcinations (in H_2_) after the deposition of Au onto supports. The use of the H_2_ calcination method in the synthesis of nanoparticles is very dangerous and cumbersome. 

Mb can reduce Au(III) to Au(0), which results in the formation of GNPs [26]. In a previous study, we demonstrated a facile Mb-mediated one-step synthetic route to prepare monodispersed GNPs. This Mb-mediated bioreduction method offers considerable advantage for creating GNPs with a narrow size distribution and a desired diameter, owing to the presence of Mb molecules, which play dual roles as both reductant and stabilizer. In this paper, the preparation of Au/Al_2_O_3_ catalyst for liquid phase glucose oxidation by the novel incipient wetness-Mb-mediated bioreduction method was investigated. 

The method is designed via an adsorption of Au(III) ions on the support followed by *in situ* bioreduction of the Au(III) ions with Mb instead of the calcination procedure (in H_2_), to avoid the agglomeration of the gold species during the calcination process. Furthermore, it has been found that these methods easily achieve high Au capture efficiency (almost 100%) by establishing a strong electrostatic interaction between Au anionic species (e.g., [AuCl_3_(OH)]^−^, [AuCl_2_(OH)_2_]^−^) in the impregnation solution and the protonated and positively-charged Al_2_O_3_ support. In our experiment, no loss of gold was observed by atomic absorption spectrophotometer during the preparation procedure. 

**Figure 1:**
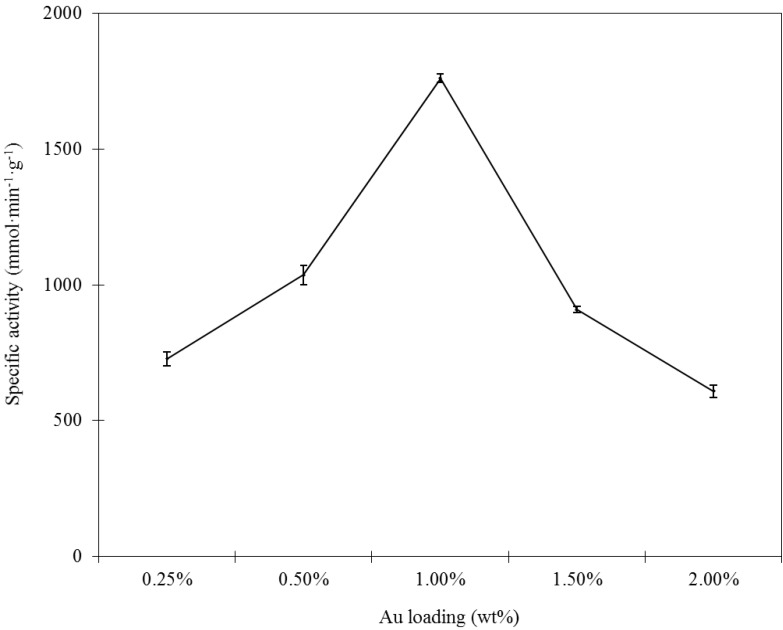
The specific activity of Au/Al_2_O_3_ catalysts with different Au loadings.

To evaluate this incipient wetness-Mb-mediated bioreduction method described in the present paper, catalysts with gold loadings in the range of 0.25 up to 2.00 wt % were prepared. The specific activity of Au/Al_2_O_3_ catalysts for glucose oxidation with H_2_O_2_ was measured. As shown in Figure 1, a strong dependence of specific activity on the gold loadings has been found. The highest specific activity was observed at a gold loading of about 1.0 wt %. Further increasing of the gold loading led to a decrease in specific activity. The catalytic performance is closely associated with both the size of GNPs and the amount of active Au sites. Higher loading can ensure sufficient active Au sites; however, larger Au particles are usually obtained at a higher Au loading. An optimum Au loading of sufficient active sites with smaller GNPs sizes is highly required. In Section 2.3, the Au particle sizes of these Au/Al_2_O_3_ catalysts were investigated by TEM. A trend of increasing particle sizes with increasing Au loading was found. Therefore, the inferior performance obtained from the low Au loading (0.25–0.5 wt %) might be ascribed to the inadequate presence of active Au sites, though smaller GNPs were acquired; while continuously increasing the Au loading to about 1.5 wt % and 2.0 wt % led to the decrease in specific activity, which was associated with the presence of larger GNPs, which was probably less catalytically active in glucose oxidation. Consequently, the optimal Au loading is 1.0%, as this was the best compromise between the Au active sites and particle sizes.

### 2.2. The Influence of Mb Overlap on the Specific Activity of Au/Al_2_O_3_ Catalyst

**Figure 2:**
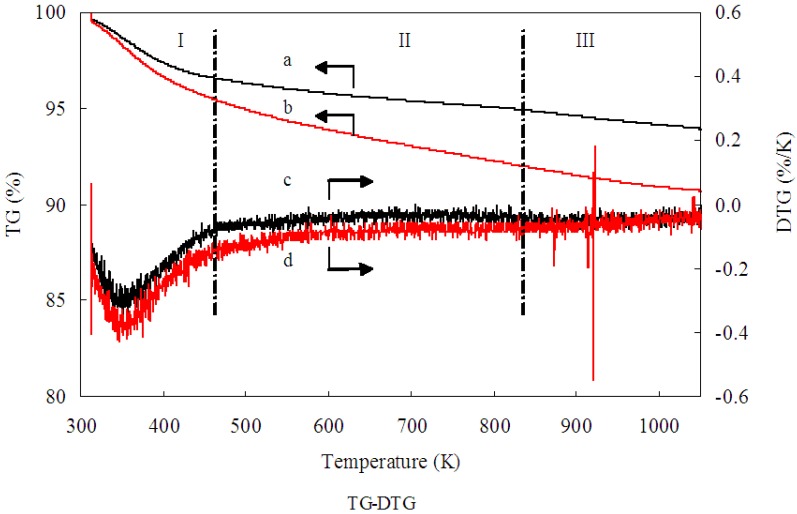
Thermogravimetric (TG) analysis profiles of (a) Al_2_O_3_; (b) Au/Al_2_O_3_ and differential thermogravimetric (DTG) analysis profiles of (c) Al_2_O_3_; (d) Au/Al_2_O_3_. Black arrow a: TG analysis profiles of Al_2_O_3_; Black arrow b: TG analysis profiles of Au/Al_2_O_3_; Black arrow c: DTG analysis profiles of Al_2_O_3_; Black arrow d: DTG analysis profiles of Au/Al_2_O_3_.

TG-DTG analysis was conducted to examine the residual Mb on the 1.0% Au/Al_2_O_3_ catalyst. Evidently, as shown in Figure 2, a weight loss of 6.2 wt % was obtained from Curve a, while a weight loss of 9.4 wt % was obtained from Curve b. The difference between Curve a and Curve b could be assigned to the decomposition of the residual Mb molecules over the Au/Al_2_O_3_ catalyst. Consequently, TG-DTG analysis indicated Mb weighed as 3.2 wt % on uncalcined 1.00 wt % Au/Al_2_O_3_ catalyst. In addition, according to the TG-DTG analysis, the removal of Mb molecules were happened in the range of 200 to 550 °C. No residual Mb molecules capped the Au/Al_2_O_3_ catalyst after 550 °C. The specific activity of Au/Al_2_O_3_ catalysts is dependent on both the particle size and the active sites of GNPs, which could not be overlapped by Mb molecules capping the surface of GNPs. The Mb molecules would overlap some active sites and, therefore, inhibited the catalytic activity toward glucose oxidation, though they acted as reductant and protective agents during the Au/Al_2_O_3_ catalyst preparation. It is well known that calcination is widely regarded as an efficient way to activate catalysts and enhance the interaction between GNPs and support. Furthermore, decomposing the Mb molecules properly through calcinations may help the exposure of the active Au surface and, thus, enhance the catalytic performance. In order to investigate the influence of Mb molecule overlap on the specific activity of Au/Al_2_O_3_ catalyst, the 1.00 wt % Au/Al_2_O_3_ catalyst was calcined under different conditions. We expect that the calcination treatment benefits the removal of a significant amount of Mb molecule residual on the Au/Al_2_O_3_ catalyst and, thus, helps the exposure of the active Au surface. The results of glucose oxidation on 1.00 wt % Au/Al_2_O_3_ catalyst that was prepared under different calcination conditions are listed in Table 1. As shown, the catalyst that was calcined at 450 °C for 2 h exhibited the highest specific activity. The inferior specific activity obtained from those who were calcined at higher temperature or for prolonged hours was likely ascribed to the agglomeration of GNPs caused by the lack of Mb protection and the excessively strong interaction between GNPs and Al_2_O_3_ support. Therefore, an appropriate calcination treatment at 450 °C for 2 h is recommended according to the results of TG-DTG analysis and the specific activity of Au/Al_2_O_3_ catalysts.

**Table 1:** Specific activity of Au/Al_2_O_3_ catalysts calcined at different temperatures and times.

Temperature	Time (h)	Specific Activity
150 °C	2 h	1203.37 ± 21.670
250 °C	2 h	1388.89 ± 40.907
350 °C	2 h	1657.55 ± 11.620
450 °C	2 h	1760.56 ± 15.95
550 °C	2 h	1360.54 ± 9.323
450 °C	1 h	1474.93 ± 20.733
450 °C	3 h	1494.77 ± 6.506
450 °C	4 h	1410.44 ± 12.642

### 2.3. TEM Observations

**Figure 3:**
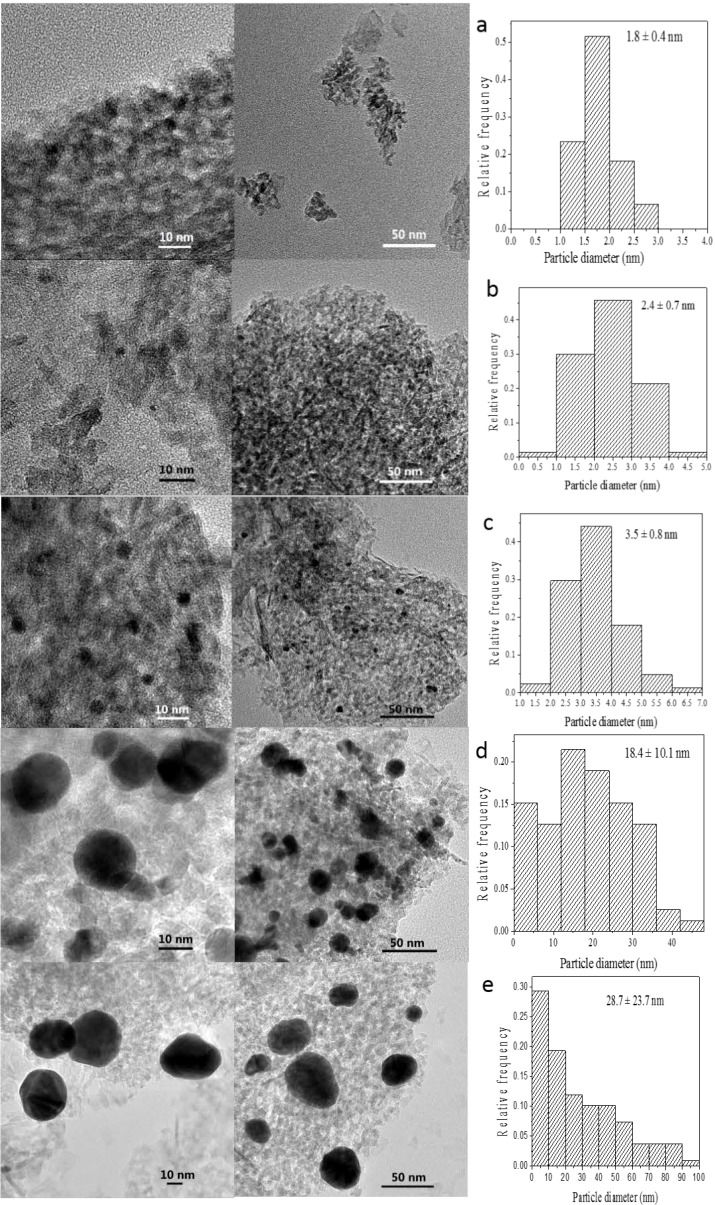
TEM images of (**a**) 0.25 wt % Au/Al_2_O_3_; (**b**) 0.50 wt % Au/Al_2_O_3_, (**c**) 1.00 wt % Au/Al_2_O_3_; (**d**) 1.50 wt % Au/Al_2_O_3_ and (**e**) 2.00 wt % Au/Al_2_O_3_.

**Figure 4:**
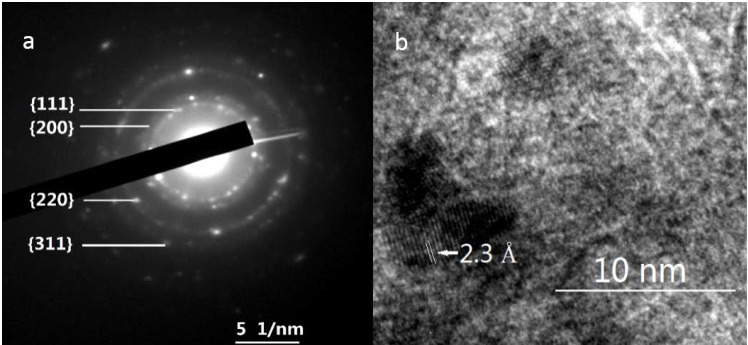
Representative TEM image of single Au nanoparticles (**a**) and their electron diffraction pattern (**b**). The diffraction spots of Rings 1, 2, 3 and 4 are due to the (111), (200), (220) and (311) reflections of the lattice planes of the facing center cube. The white arrow in (**b**) indicates that the d-spacing of the adjacent lattice of the GNPs was 2.3 Å.

The size of GNPs is very important for the catalytic performance of supported nanogold catalysts. Analysis of the morphology and particle size of the supported GNPs by TEM should help determine whether a correlation exists among gold loading, specific activity and particle size. TEM images of several selected catalysts with various gold contents prepared by the incipient wetness-Mb-mediated bioreduction method are shown in Figure 3. Histograms of their size distributions are also given in the right of Figure 3. As illustrated, TEM characterization reveals that all of the samples presented nearly spherical GNPs with different mean diameter values from 1.9 to 16.6 nm that are well dispersed on the support. An increase in the gold loading leads to an increase in particle size. Combining the results shown in Figure 1 and Figure 3, the optimal Au loading is 1.0%, as this was the best compromise between the Au active sites and particle sizes. The selected-area electron diffraction (SAED) pattern with four bright circular rings corresponding to the (111), (200), (220) and (311) planes exhibited the crystalline nature of AuNPs (Figure 4A) representative TEM image of single AuNP shows that the distance between the two layers is 0.23 nm. The layers implied the crystalloid arrangement of the Au atomic layer (Figure 4B).

### 2.4. XPS Analysis

**Figure 5:**
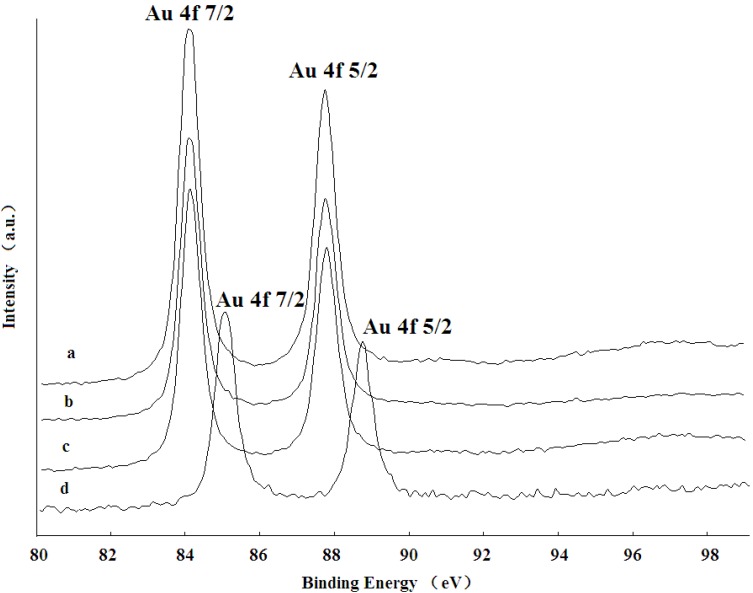
XPS spectra of Au 4f regions recorded for the (a) calcined catalyst before reaction; (b) bioreduced catalyst without calcinations; (c) calcined catalyst after reaction and (d) pure support-absorbed chloroauric acid.

As shown in Figure 5, the XPS spectrum of the catalyst precursor (pure Al_2_O_3_ support adsorbed chloroauric acid) with an Au 4f 7/2 signal around 85.3 eV and 4f 5/2 around 89.0 eV confirmed that Au species had not been reduced yet (curve d). However, the XPS spectra of the fresh Au/Al_2_O_3_ catalyst before the reaction (Curve a, calcined at 450 °C) and un-calcined Au/Al_2_O_3_ catalyst (Curve b) showed the similar Au 4f 7/2 signal with a lower position at 83.7 eV and the Au 4f 5/2 signal at 87.5 eV, which indicated that Au ions were reduced to the metallic phase and that the phase was maintained after calcinations.

The Au 4f core level spectrum measured from the Au/Al_2_O_3_ catalyst is fitted well by a single doublet consisting of Au 4f 7/2 and Au 4f 5/2 peaks arising from spin-orbital coupling and separated by 3.7 eV. This metallic gold was the only gold species observed from the catalyst sample, suggesting complete reduction by Mb. Besides, the XPS spectra for the Au/Al_2_O_3_ catalyst before the reaction (Curve a) and after the reaction (Curve c) was almost identical, suggesting that supported GNPs were relatively stable and that Au maintained the Au(0) state after the reaction.

### 2.5. XRD Analysis

**Figure 6:**
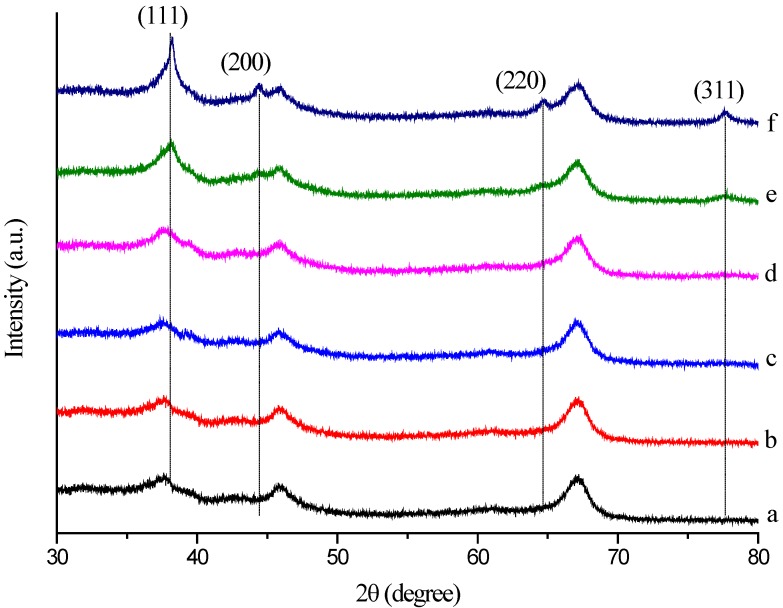
XRD pattern of the crystalline AuNPs, which were formed by Mb-mediated bioreduction (a) Al_2_O_3_; (b) 0.25 wt % Au/Al_2_O_3_; (c) 0.50 wt % Au/Al_2_O_3_; (d) 1.00 wt % Au/Al_2_O_3_; (e) 1.50 wt % Au/Al_2_O_3_; (f) 2.00 wt % Au/Al_2_O_3_.

X-ray diffraction was also used to confirm the crystalline nature of the particles. The crystalline phases of the Au/Al_2_O_3_ catalyst were reflected by the powder XRD patterns in Figure 6. The XRD pattern exhibited two diffraction peaks appearing at 2 θ = 45.9° and 66.9°, which can indicate that the carrier was composed by γ-Al_2_O_3_. The diffraction peaks at 2θ = 38.2° (111), 44.4° (200), 64.6° (220) and 77.7° (311) obtained are identical to those reported for the standard gold metal (Au (0)) (Joint Committee on Powder Diffraction Standards (JCPDS), Swarthmore, PA, USA). Thus, the XRD pattern suggests that the GNPs were essentially crystalline. The slight shift in the peak positions may be due to the presence of Mb molecules in the crystal structure. Furthermore, as shown, the intensity of the four diffraction peaks is much lower and even disappeared at a low Au loading and increased with increasing Au loading. These observations indicated that the gold nanoplates formed are of smaller dimensions and well dispersed at a low Au loading. The XRD pattern provided strong evidence in favor of the TEM images for the presence of gold nanocrystals. The size of the GNPs was also estimated by the Scherrer method [27]. The average size of the GNPs according to the Scherrer equation was determined to be about 18.58 nm ± 0.60 nm at 1.50 wt % Au loading of Al_2_O_3_ and 22.67 nm ± 1.89 nm at 2.00 wt % Au loading of Al_2_O_3_. These results were in good accordance with the particle size obtained from the TEM image.

### 2.6. FTIR Analysis

**Figure 7:**
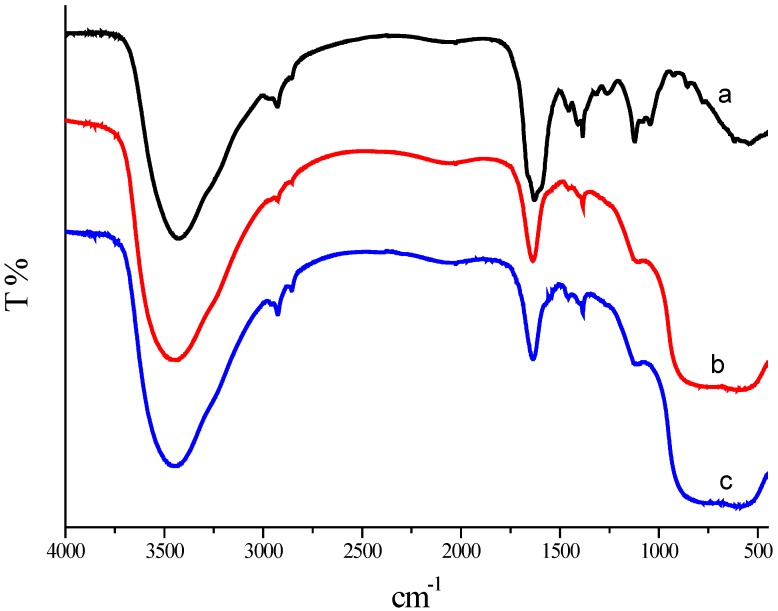
FTIR spectra of the Mb (Curve a); the un-calcined Au/Al_2_O_3_ catalyst synthesized by the reduction of chloroauric acid with Mb (Curve b); and calcined 1.00 wt % Au/Al_2_O_3_ catalyst synthesized by the reduction of chloroauric acid with Mb (Curve c).

FTIR analysis was performed for the characterization of Mb and the synthesized Au/Al_2_O_3_ catalyst. The experiments revealed the presence of vibration bands centered at 2928.97, 2853.15, 1630.11, 1456.66, 1409.75, 1261.00, 1044.14 cm^−1^ along with an intense broad band at 3433.63 cm^−1^ (Figure 7). The broad intense band at about 3433.63 cm^−1^ results from stretching vibrations of H-bonded hydroxyl groups and the N–H stretching of secondary amides. Weaker bands at 2928.97 and 2853.15 cm^−1^ can be attributed to the C–H stretching of aliphatic CH_3_ and CH_2_. The band at 1630.11 and 1456.66 cm^−1^ may result from C=O stretching of the carboxyl group and ketones, and 1261.00 cm^−1^ may be assigned to the amide stretching. The band at around 1044.14 cm^−1^ indicated C–O stretching. The peak at 1409.75 cm^−1^ may be assigned to the symmetric stretching of the carboxyl side groups in the Mb molecules. 

These bands clearly implied the presence of peptides on the GNPs surface. The slight shift in the stretching frequency results from significant interaction between the Mb molecules and the GNPs surface. These Mb molecules act as surface coating molecules, which prevent the internal agglomeration of GNPs. The FTIR spectra of the Au/Al_2_O_3_ catalyst before and after appropriate calcination treatment at 450 °C for 2 h did not show any significant changes. The band assigned to C=O stretching is visible before and after appropriate calcination treatment at 450 °C for 2 h. This means there were some Mb molecules remaining at the surface of the GNPs. This result is in accordance with the TG analysis. According to the TG-DTG analysis, the removal of Mb molecules happened in the range of 200 to 550 °C. No residual Mb molecules capped the Au catalysts after 550 °C. An outright elimination of Mb may result in the sintering of Au particles. Therefore, an appropriate calcination treatment at 450 °C for 2 h is recommended followed by the results of catalytic performance and was confirmed by the results of TG, TEM and FTIR analysis.

### 2.7. Catalyst Durability

**Figure 8:**
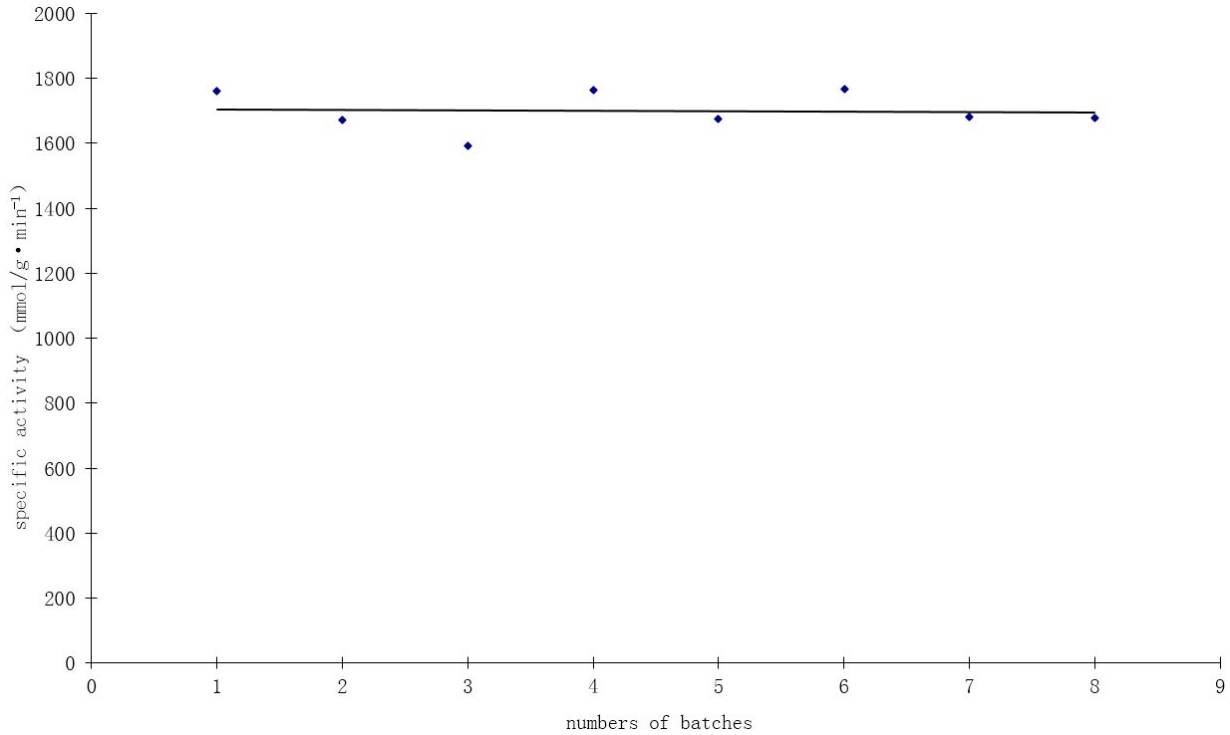
Recycling test of the 1.0 wt % Au/Al_2_O_3_ catalyst in the oxidation of glucose.

Durability is an important parameter of heterogeneous catalysts. Recycle tests were conducted to assess the durability of the 1.0 wt % Au/Al_2_O_3_ catalyst prepared by the incipient wetness-Mb-mediated bioreduction method. The used catalyst was recovered by filtration and washed with deionized water, then dried and reused for another reaction under the same conditions. As shown in Figure 8, high catalyst activity of about 1700 mmol/g·min^−1^·Au^−1^ was obtained at mild reaction conditions (40 °C, pH 9.5) and atmospheric pressure. The specific activity remained fairly constant, with some fluctuations during eight consecutive cycles, indicating the remarkable stability and durability of the bioreduction Au catalyst. 

TEM observations of this catalyst used for eight repeated batches showed no significant change of Au size after the evaluation test, indicating the potential stabilizing role of Mb. Elemental analysis before the first batch and after the last batch showed no loss of gold during the experiment. These findings reveal that the catalysts prepared by the incipient wetness-Mb-mediated bioreduction method have excellent catalytic stability. Thielecke *et al.* [28] has reported that A 0.3% Au/Al_2_O_3_ catalyst prepared by the incipient wetness method showed an activity of 150 mmol/min·g^−1^ within its 110 days of continuous-flow glucose oxidation operation. Baatz *et al.* [14] has also reported that A 0.3% Au/Al_2_O_3_ catalyst prepared by incipient wetness showed an activity of 550 mmol/min·g^−1^ for liquid-phase glucose oxidation, with oxygen as the oxidizing agent. 

In the present work, we demonstrate that the incipient wetness-Mb-mediated bioreduction method is a promising alternative method of preparing Au/Al_2_O_3_ catalysts for glucose oxidation. A 1.0% Au/Al_2_O_3_ catalyst prepared by the method showed an activity of 1700 mmol/min·g^−1^ for liquid-phase glucose oxidation with H_2_O_2_ as the oxidizing agent. We proposed that Mb, as both the protecting and reducing agent, combined on the surface of the AuNPs, is able to prevent the leaching of Au and yet, does not offer overprotection; thereby contributing to the excellent durability of the bioreduction Au/Al_2_O_3_ catalysts.

## 3. Experimental Section

### 3.1. Materials

Chloroauric acid (HAuCl_4_, 49.8 wt % Au) was purchased from Shanghai July Chemical Co., Ltd. (Shanghai, China) and used directly without pretreatment. *Methylosinus trichosporium* 3011 was obtained from the Institute of Microbiology and Virology (Kiev, Ukraine) [29].

### 3.2. Preparation of Mb

*Methylosinus trichosporium* 3011 was grown in a 5-L bioreactor containing 3 L copper-deficient mineral salt medium, as previously described [29]. Methanol was added to 0.2% (*v*/*v*) and supplied on-line to keep the same concentration. Cells were grown at 28–30 °C at an agitation rate of 250–300 rpm. Ambient air was bubbled through the fermenter continuously at 0.5–0.8 L/min. The cultures were grown to the stationary phase for Mb production.

Mb from the spent medium of *Methylosinus trichosporium* 3011 was isolated as previously described for *Methylococcus capsulatus* Bath by Choi *et al.* [30]. The cells were removed by centrifugation at 10,000× *g* for 30 min. The supernate was loaded onto a 2.5 × 20-cm Diaion HP-20 column (Mitsubishi Chemical Holdings, Tokyo, Japan). The bound Mb was washed with two column volumes of H_2_O and eluted with 40% methanol: 60% H_2_O. The eluant was lyophilized for concentration and storage. The freeze-dried samples following chromatography on Diaion HP-20 columns were the source of Mb used in this study. The amount of Mb in the sample was quantified according to Xin *et al.* [26]. The concentration of Mb was measured by spectrophotometry using Chrome Azure S [26].

### 3.3. Preparation of Au/Al_2_O_3_ Catalyst

Al_2_O_3_ (particle size: 100 nm; specific surface area: >200 m^2^/g; pore volume = 0.45 mL /g) was used as the support, and HAuCl_4_ was used as the gold precursor. A typical procedure is as follows. The required amount of HAuCl_4_ was dissolved in a volume of deionized water corresponding to the pore volume of the support. The solution was added drop-wise to the Al_2_O_3_ support during intensive mixing. After complete addition of the solution, the Al_2_O_3_ support became slightly wet. The resulting precursor was dried overnight at 80 °C and then was added to the 0.1 mM aqueous solutions of Mb. 

The Au precursors were reduced *in situ* on the support by Mb, which serves dual roles as reducing and stabilizing agents. Further on, the catalyst was filtered, subsequently washed thoroughly with deionized water, until no more chloride was observed, dried at 80 °C and under vacuum for 6–12 h, and finally, the catalyst was activated by calcination in air at 150–550 °C for 1–4 h. The amount of gold on the catalysts is always given as a weight fraction (wt %). All catalysts were used for glucose oxidation directly after preparation.

### 3.4. Characterization of Au/Al_2_O_3_ Catalysts

Transmission electron microscopy (TEM) images of the Au/Al_2_O_3_ catalysts were obtained on a JEOL JEM-2100F electron microscope (JEOL Ltd., Tokyo, Japan) equipped with the selected area electron diffraction pattern (SAED). An acceleration voltage of 200 kV was used to determine gold particle size and its distribution. The samples for TEM were prepared on carbon-coated copper grids followed by solvent evaporation under vacuum. Particle sizes were determined by the ImageJ Java program (National Institutes of Health, Bethesda, MD, USA) using the “Particle Analyzer” function. The mean diameters of gold metal particles for each catalyst were determined by counting over 150 particles in TEM images taken with a medium magnification.

Thermogravimetric (TG) analysis was done to ascertain the content of Mb on the Au/Al_2_O_3_ catalysts, which was carried out on a PerkinElmer Pyris-6 (Perkinelmer, Waltham, MA, USA) Thermogravimetric Analyzer under flowing air atmosphere at a heating rate of 10 °C·min^−1^ from 40 to 800 °C.

Powder X-ray diffraction (XRD) measurements were recorded on a Thermo Fisher K-Alpha X-ray diffractometer (PANalytical B.V., Eindhoven, The Netherlands) that was operated at a voltage of 40 kV and a current of 40 mA with Cu Kα radiation with λ of 0.15406 nm). The scanning was done in the region of 2θ from 30° to 80°.

X-ray photoelectron spectroscopy (XPS) was performed using a K-Alpha X-ray photoelectron spectroscopy (Thermo Fisher Scientific, Waltham, MA, USA). The valence states of elements were analyzed by using K-Alpha X-ray photoelectron spectroscopy (Thermo Fisher Scientific, Waltham, MA, USA). The samples for XPS were prepared by adding a 0.2-mL dispersed solution of Au/Al_2_O_3_ catalyst or Al_2_O_3_ carrier onto a glass plate and allowing water to completely evaporate. The Au/Al_2_O_3_ samples for XPS were prepared by centrifuging the dispersed solution of Au/Al_2_O_3_ at 4000 rpm for 10 min. The pellet was redispersed with deionized water three times to get rid of the unattached Mb molecules.

The FTIR analyses of Mb, calcined Au/Al_2_O_3_ catalyst and un-calcined Au/Al_2_O_3_ catalyst samples were performed on a PerkinElmer 100 FTIR Spectrometer operating at a resolution of 4 cm^−1^ over 4000–450 cm^−1^. The Mb sample was freeze-dried, and the Au/Al_2_O_3_ sample was redispersed with deionized water three times to get rid of the unattached Mb molecules that are not capping ligands for the gold nanoparticles. Additionally, 0.2–0.5 mg of the dried sample material were ground with 300 mg dried KBr and pressed into a pellet. A background spectrum was recorded with a pellet containing 300 mg of KBr.

### 3.5. Catalytic Activity Measurements

Glucose oxidation catalyzed by the Au/Al_2_O_3_ catalyst was carried out using H_2_O_2_ as the oxidant at 40 °C, as previously described [7]. A known amount of the catalyst (0.2 g) dispersed in H_2_O (0.6 mL) was added into 5 wt % aqueous glucose solution (22 mL) in a 50-mL three-necked round-bottomed flask equipped with a thermostat, a magnetic stirrer, a peristaltic pump for alkali (0.5 M NaOH) supply, a glass combination electrode filled with 1 M aqueous solution of KNO_3_ and a reflux condenser. The pH was adjusted to 9.5 by adding aqueous NaOH at the initial time. In order to initiate and proceed with the reaction, 30 wt % H_2_O_2_ was added. The reaction was monitored by maintaining the pH at 9.5 through the titration with 0.5 M NaOH aqueous solution. The addition of H_2_O_2_ (30 wt % solution) was carried out manually and in constant portions during the reaction course at the consumed NaOH solution volume, which equals a glucose conversion of approximately 3%–5%. Unless otherwise stated, a total of 1.1 equivalent H_2_O_2_ in relation to glucose and 0.2 g Au/Al_2_O_3_ catalyst were used. Samples for analyses were periodically drawn from the reaction mixture. Conversion of glucose to sodium gluconate was calculated from the total amount of sodium gluconate yield (Section 2.6). Because of the different gold contents of the catalysts used, the specific activity of the catalyst was used to compare catalysts with different gold contents, which was calculated as the activity of the catalyst at a <10% conversion of the substrate. 

The catalyst’s durability was investigated by repeated batches of glucose oxidation. All reactions were carried out until 100% conversion was reached. The catalyst was filtered from the reaction mixture after one batch and washed with deionized water and dried overnight at 70 °C before reusing it in the following run. 

### 3.6. Gluconic Acid Measurement

The gluconic acid product was measured with a gluconic acid-specific colorimetric assay according to a previously reported procedure [31]. Au/Al_2_O_3_ catalyzes the oxidation of glucose to gluconic acid, and the generated gluconic acid reacts with hydroxylamine; after the addition of Fe(III), the red complex hydroxamate-Fe(III) with a maximum absorbance at 505 nm is formed. Briefly, 250 µL of Solution 1 (5.00 mM EDTA and 0.15 mM Et_3_N in water) and 25 µL of Solution 2 (3.00 M NH_2_OH in water) were added to the catalytic reaction solution, and the mixture was allowed to react for 15 min. Finally, 125 mL of Solution 3 (1.00 M HCl, 0.10 M FeCl_3_ and 0.25 M CCl_3_COOH in water) were added to the reaction medium, and the reaction was allowed to proceed for 5 min.

## 4. Conclusions

In heterogeneous catalysts, metal NPs have usually been stabilized or immobilized by being supported on thermally-stable Al_2_O_3_. From ecologic and economic standpoints, the incipient wetness-Mb-mediated bioreduction method should be favored, because of no gold loss and wastewater production. Further on, smaller equipment and fewer process steps are needed to accomplish catalyst preparation. During the *in situ* reduction with Mb, chloride likely is discharged by the Mb solution. This interpretation of the fact that the 1.0 wt % Au/Al_2_O_3_ catalyst was prepared by this method means that it could obtain a mean Au diameter as small as 4 nm. Furthermore, the method is advantageous owing to the fact that the gold is already reduced, shortening the catalyst preparation time. Thus, the incipient wetness-Mb-mediated bioreduction method can be regarded as a preferable method for the preparation of Au/Al_2_O_3_ catalyst.

In summary, we have demonstrated an excellent and efficient incipient wetness-Mb-mediated bioreduction method of preparing Au/Al_2_O_3_ catalyst for liquid-phase glucose oxidation with aqueous H_2_O_2_, which exhibited both high specific activity and durability. We believe that the method of catalyst preparation is promising for industrial applications.
